# Crystal structure of the Ba^II^-based Co^II^-containing one-dimensional coordination polymer poly[[aqua{μ_4_-2,2′-[(4,10-dimethyl-1,4,7,10-tetra­aza­cyclo­dodecane-1,7-di­yl)bis(methylidene)]bis­(4-oxo-4*H*-pyran-3-olato)}­perchloratocobaltbarium] perchlorate]

**DOI:** 10.1107/S2056989017015638

**Published:** 2017-11-03

**Authors:** Paola Paoli, Eleonora Macedi, Patrizia Rossi, Luca Giorgi, Mauro Formica, Vieri Fusi

**Affiliations:** a Department of Industrial Engineering, University of Firenze, via Santa Marta 3, I-50139 Firenze, Italy; b Department of Pure and Applied Sciences, Lab of Supramolecular Chemistry, University of Urbino, via della Stazione, 4, I-61029 Urbino, Italy

**Keywords:** crystal structure, coordination polymer, barium, cobalt, macrocycle

## Abstract

The title compound, {[Ba{Co(H_-2_ **L1**)}(ClO_4_)(H_2_O)]ClO_4_}_*n*_, **L1 =** 4,10-bis­[(3-hy­droxy-4-pyron-2-yl)meth­yl]-1,7-dimethyl-1,4,7,10-tetra­aza­cyclo­dodeca­ne, is a one-dimensional coordination polymer. The asymmetric unit consists of a {Ba[Co(H_–2_ **L1**)](ClO_4_)(H_2_O)}^+^ cationic fragment and a non-coordinating ClO_4_^−^ anion. In the neutral [Co(H_–2_ **L1**)] moiety, the cobalt ion is hexa­coordinated in a trigonal–prismatic fashion by the surrounding N_4_O_2_ donor set. The Ba^2+^ ion is nine-coordinated and exhibits a distorted [BaO_9_] monocapped square-anti­prismatic geometry, the six oxygen atoms coming from three distinct [Co(H_–2_ **L1**)] moieties, while the remaining three vertices are occupied by the oxygen atoms of a bidentate perchlorate anion and a water mol­ecule. A barium–μ_2_-oxygen motif develops along the *a* axis, connecting symmetry-related dinuclear Ba^II^–Co^II^ cationic fragments in a wave-like chain, forming a one-dimensional metal coordination polymer. Non-coordinating ClO_4_^−^ anions are located in the space between the chains. Weak C—H⋯O hydrogen bonds involving both coordinating and non-coordinating perchlorate anions build the whole crystal architecture. To our knowledge, this is the first example of a macrocyclic ligand forming a Ba^II^-based one-dimensional coordination polymer, containing Co^II^ ions surrounded by a N_4_O_2_ donor set.

## Abstract 2

A barium-μ_2_-oxygen motif develops along the *a* axis, connecting symmetry-related dinuclear Ba^II^–Co^II^ cationic fragments in a wave-like chain, forming a one-dimensional metal coordination polymer. Non-coordinating ClO_4_^−^ anions are located in the space between the chains in this first example of a macrocyclic ligand forming a Ba^II^-based one-dimensional coordination polymer, containing Co^II^ ions surrounded by a N_4_O_2_ donor set.

## Chemical context   

Metal coordination polymers (CPs) have witnessed continuous growth, owing to their fascinating structural diversity in terms of architecture and topology and also their numerous potential applications, such as gas storage (Banerjee *et al.*, 2016 ▸; Fracaroli *et al.*, 2014 ▸; Sumida *et al.*, 2012 ▸; Suh *et al.*, 2012 ▸), chemical sensing (Campbell *et al.*, 2015 ▸; Hu *et al.*, 2014 ▸; Wang *et al.*, 2013 ▸; Kreno *et al.*, 2012 ▸), catalysis (Chughtai *et al.*, 2015 ▸; Mo *et al.*, 2014 ▸; Yoon *et al.*, 2012 ▸; Liu, Xuan *et al.*, 2010 ▸) and so forth. Recently, the inter­est in alkaline-earth metal ion-based CPs has been growing due to their unusual advantages such as low toxicity, wide distribution and low cost, which are of benefit for applications in the field of materials science (Raja *et al.*, 2014 ▸; Foo *et al.*, 2012 ▸, 2013 ▸; Xiao *et al.*, 2012 ▸).

According to a Cambridge Structural Database (CSD, Version 5.38, May 2017; Groom *et al.*, 2016 ▸) search, alkaline-earth metal-based CPs are less common compared to the reported transition metal and rare-earth metal CPs (Cai *et al.*, 2017 ▸). Indeed, the study of alkaline-earth–metal systems is limited by challenges in the synthesis (Lian *et al.*, 2016 ▸; Douvali *et al.*, 2015 ▸; Mali *et al.*, 2015 ▸; Chakraborty *et al.*, 2014 ▸; Zhang, Huang *et al.*, 2012 ▸; Liu, Tsao *et al.*, 2010 ▸), the main reason being the variable coordination numbers (the most preferred coordination numbers are six for magnesium, six to eight for calcium, and six to twelve for strontium and barium), which lead to uncontrolled coordination geometries around the metal centre (Cai *et al.*, 2016 ▸; Feng *et al.*, 2015 ▸; Shi *et al.*, 2015 ▸; Zheng *et al.*, 2015 ▸; Jia *et al.*, 2014 ▸; Zhang, Yuan *et al.*, 2013 ▸; Smith *et al.*, 2013 ▸; Zhai *et al.*, 2013 ▸; Zhang, Guo *et al.*, 2013 ▸; Deng *et al.*, 2012 ▸; Foo *et al.*, 2012 ▸; Xiao *et al.*, 2012 ▸; Xie *et al.*, 2012 ▸; Zhang, Luo *et al.*, 2012 ▸; Jing *et al.*, 2010 ▸; Zhang *et al.*, 2010 ▸; Li *et al.*, 2009 ▸).

Besides, the ability of a system to bind alkaline-earth metal ions in aqueous solution is highly desirable and can be achieved thanks to the presence of oxygenated ligands and the preorganization of the receptor, which satisfies the need for a high coordination number without specific coordination requirements.

Ligand **L1** {4,10-bis­[(3-hy­droxy-4-pyron-2-yl)meth­yl]-1,7-dimethyl-1,4,7,10-tetra­aza­cyclo­dodeca­ne} is a Maltol-based macrocycle (Amatori *et al.*, 2012 ▸) and is able to form discrete heteropolynuclear complexes. It has already proved to able form a Co^II^ species (Borgogelli *et al.*, 2013 ▸) that is able to bind hard metal ions such as *Ln* ^III^ (*Ln* = Gd, Eu) and Na(I). In the case of *Ln* ^III^ ions, heterotrinuclear Co^II^–*Ln* ^III^–Co^II^ systems form, where the Co^II^ cation preorganizes the system and two Co^II^ species are involved in the coordination of one *Ln* ^III^ ion (Benelli *et al.*, 2013 ▸; Rossi *et al.*, 2017 ▸). In the case of the alkaline ion, a heterodinuclear complex forms, involving only one Co^II^ species (Borgogelli *et al.*, 2013 ▸).

Herein we present a Ba^II^–Co^II^ heterodinuclear metal coordination compound of **L1**, where a one-dimensional wave-like infinite array of barium ions bridges the [Co(H_–2_ **L1**)] moieties through a barium–μ_2_-oxygen motif. This is the first time that **L1** has proven able to form a coordination polymer and, to our knowledge, this is the first example of a macrocyclic ligand forming a Ba^II^-based 1D-CP containing Co^II^ ions surrounded by an N_4_O_2_ donor set.
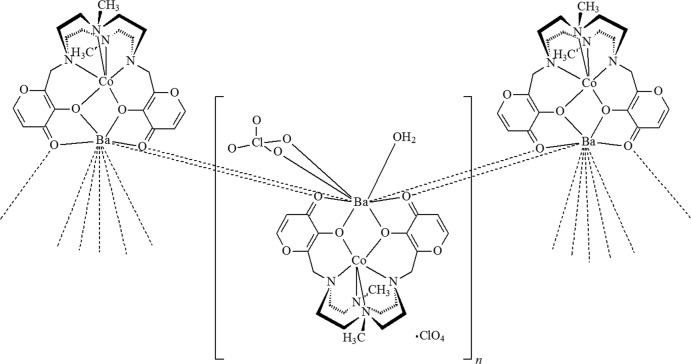


## Structural commentary   

**Figure 1:**
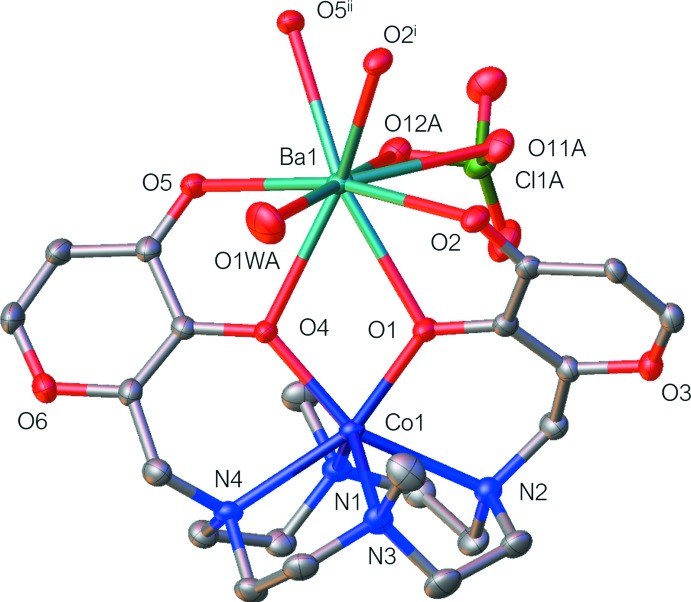
The mol­ecular structure of the {Ba[Co(H_–2_ **L1**)](ClO_4_)(H_2_O)}^+^ cationic fragment, with the atom labelling and 30% probability displacement ellipsoids. Only one component of the disordered perchlorate anion and water mol­ecule is shown. H atoms have been omitted for clarity. Symmetry codes: (i) −*x* + 1, −*y*, −*z*; (ii) −*x* + 2, −*y*, −*z*.

The title compound is the Ba^II^-based Co^II^-containing 1D-CP of **L1** of formula {{Ba[Co(H_–2_ **L1**)](ClO_4_)(H_2_O)}·ClO_4_}_*n*_ and crystallizes in the monoclinic system in space group *P*2_1_/*n*, with a {Ba[Co(H_–2_ **L1**)](ClO_4_)(H_2_O)}^+^ cationic fragment (Fig. 1 ▸) and a (ClO_4_)^−^ anion in the asymmetric unit.

**Figure 2:**
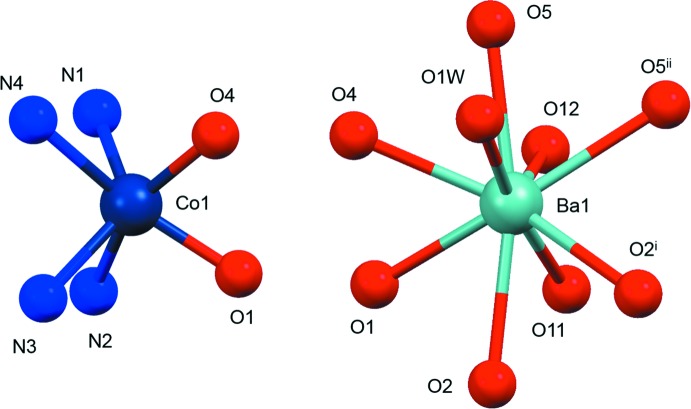
Coordination polyhedra around cobalt (left) and barium (right) ions. [Symmetry codes: (i) −*x* + 1, −*y*, −*z*; (ii) −*x* + 2, −*y*, −*z*.]

**Figure 3:**
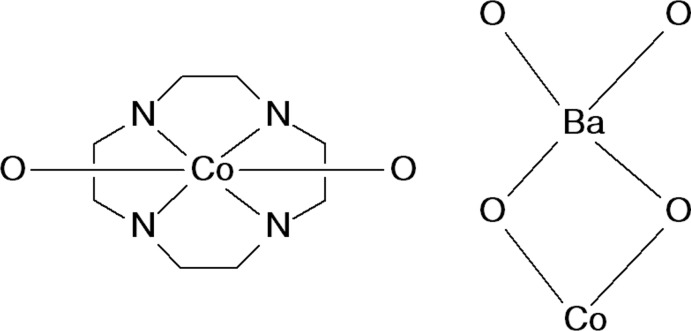
Fragments searched in the CSD.

In the neutral [Co(H_-2_ **L1**)] moiety, the Co^2+^ ion is hexa­coordinated and exhibits a distorted trigonal–prismatic geometry (Muetterties & Guggenberger, 1974 ▸), where the cobalt ion is surrounded by four nitro­gen atoms of the macrocyclic base and two deprotonated hydroxyl oxygen atoms provided by both maltolate rings of the ligand. In the distorted trigonal prism, the O1,N2,N3/O4,N1,N4 atoms define the two triangular faces, which are parallel within 12.51 (11)° (Fig. 2 ▸). The cobalt ion is displaced 1.0971 (5) Å above the mean plane described by the four nitro­gen atoms of the tetra­aza­macrocycle [maximum deviation of 0.068 (4) Å for N3] and falls, together with the Co—N(CH_3_) and Co—O bond distances (Table 1 ▸), in the expected range for Co-[12]aneN_4_ complexes where the cobalt ion is hexa­coordinated with a N_4_O_2_ donor set (Fig. 3 ▸, left). The Co—N(Maltol) bond distances, instead, are longer (Table 1 ▸) than the Co—N(CH_3_) ones and longer with respect to those reported for other Co–**L1** complexes [Co—N(Maltol): range 2.26–2.44; Co—N(CH_3_) range: 2.13–2.19; Benelli *et al.*, 2013 ▸; Borgogelli *et al.*, 2013 ▸; Rossi *et al.*, 2017 ▸].

The conformation of the [12]aneN_4_ macrocycle is the usual [3333]C-corners one (Meurant, 1987 ▸) with the *trans* nitro­gen distances in agreement with those reported in the CSD for this conformation type, but the N2⋯N4 distance being longer than the N1⋯N3 one by 0.26 Å (Table 1 ▸), as found only in 36% of cases. This is probably due to the fact that the Maltol units linked to atoms N2 and N4 are involved in chelate six-membered rings, which stiffen the system and force those nitro­gen atoms to move farther apart.

The two maltolate rings are almost orthogonal to each other (dihedral angle between ring mean planes about 71°); both rings form similar angles (about 55°) with the mean plane N1,N2,N3,N4. The dimensions of the binding area defined by the four oxygen donor atoms of the ligand, as roughly estimated by the distances separating the opposite O1⋯O5 and O2⋯O4 atoms, are quite similar (about 4.5 Å).

Tha Ba^2+^ ion is nine-coordinated and exhibits a distorted [BaO_9_] monocapped square-anti­prismatic geometry (Guggenberger & Muetterties, 1976 ▸), Fig. 2 ▸, where the barium cation is surrounded by six oxygen atoms from three distinct [Co(H_–2_ **L1**)] moieties {four from two maltolate groups of a moiety and two from the carbonyl groups belonging to two distinct symmetry-related moieties, O2^i^ and O5^ii^ [symmetry codes: (i) −*x* + 1, −*y*, −*z*; (ii) −*x* + 2, −*y*, −*z*]}, an oxygen atom of a disordered water mol­ecule and two oxygen atoms of a disordered perchlorate anion, the latter acting as a bidentate ligand (Fig. 1 ▸). In the distorted monocapped square anti­prism, the O5 oxygen atom caps the O5^ii^, O4, O1*W*, O12 face (Fig. 2 ▸, right). All bond distances (Table 1 ▸) are in agreement with data found in the CSD.

The Ba^2+^ and Co^2+^ cations are located 3.9799 (7) Å apart from each other, the line connecting them being normal to the mean plane described by the four nitro­gen atoms of the macrocycle [angle value: 87.59 (7)°; Fig. 1 ▸]. As for the bridged Co–O–Ba moiety (Fig. 3 ▸, right), while the Ba—O and Co—O bond distances and the Ba⋯Co distance are in agreement with those found in the CSD, the corresponding Ba—O—Co angles (Table 1 ▸) are outside the observed range (89.5–111.4°).

## Supra­molecular features   

**Figure 4:**
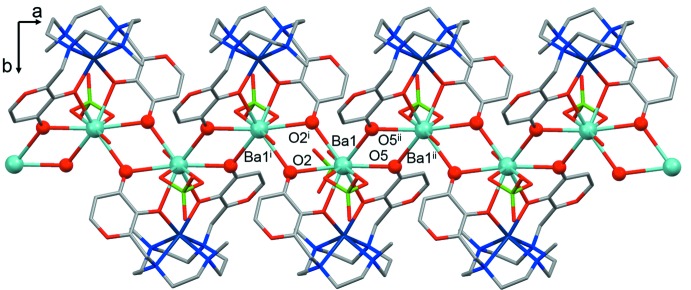
Wave-like one-dimensional Ba^II^-based coordination polymer that develops along the *a* axis. The oxygen and barium atoms belonging to the barium–μ_2_-oxygen motif are depicted in ball and stick mode. Only one component of the disordered perchlorate anion and water mol­ecule is shown. H atoms and the non-coordinating ClO_4_^−^ anions have been omitted for clarity. [Symmetry codes: (i) −*x* + 1, −*y*, −*z*; (ii) −*x* + 2, −*y*, −*z*.]

The title compound forms wave-like chains with a repeating unit comprising a dinuclear Ba^II^–Co^II^ cationic fragment with associated coordinating water mol­ecules and perchlorate ions (Fig. 4 ▸). Non-coordinating ClO_4_^−^ anions are located in the space between the chains.

A barium–μ_2_-oxygen motif develops along the *a* axis, the angle between the two mean planes formed by atoms Ba, O2, Ba^i^ and O2^i^ and atoms Ba, O5, Ba^ii^ and O5^ii^ is about 40° [symmetry codes: (i) −*x* + 1, −*y*, −*z*; (ii) −*x* + 2, −*y*, −*z*], Fig. 4 ▸. The Ba—O bond distances, the O⋯O and Ba⋯Ba distances and the Ba—O—Ba angle values within each plane and the Ba^i^⋯Ba^ii^ distance (Table 1 ▸) are in agreement with data reported in the CSD.

**Figure 5:**
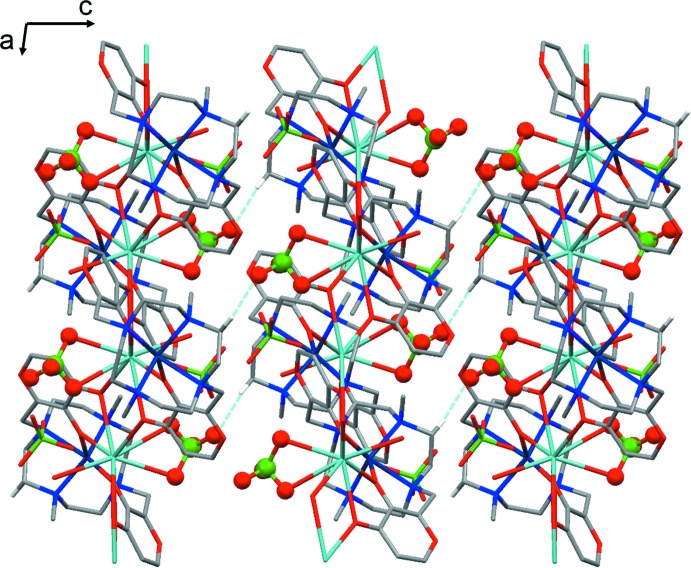
Adjacent CPs connected *via* hydrogen bonds involving the coordinating ClO_4_^−^ anions as viewed along the *b* axis. The coordinating ClO_4_^−^ anions are depicted in ball and stick mode. Hydrogen bonds are depicted as light-blue dotted lines. Only H atoms involved in the C—H⋯O inter­actions and only one component of the disordered perchlorate anion and water mol­ecule are shown.

Weak C—H⋯O hydrogen bonds (Desiraju & Steiner, 1999 ▸) involving both coordinating and non-coordinating perchlorate anions build the whole crystal architecture (Table 2 ▸). Distinct 1D-CPs are held together by weak C—H⋯O inter­actions between the coordinating perchlorate anions belonging to a CP and methyl­ene hydrogen atoms belonging to the adjacent CPs (Fig. 5 ▸).

**Figure 6:**
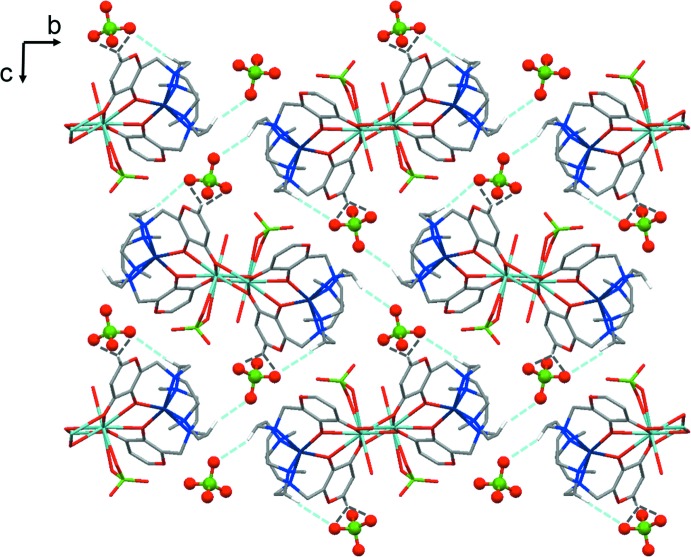
Crystal packing of the title compound as viewed along the *a* axis. The non-coordinating ClO_4_^−^ anions are depicted in ball and stick mode. Hydrogen bonds involving the non-coordinating ClO_4_^−^ anion and two {Ba[Co(H_–2_ **L1**)](ClO_4_)(H_2_O)}^+^ cationic fragments on the same plane are depicted in light-blue dotted lines. Hydrogen bonds involving the non-coordinating ClO_4_^−^ anion and a {Ba[Co(H_–2_ **L1**)](ClO_4_)(H_2_O)}^+^ cationic fragment out of plane (symmetry operation *x* − 1, *y*, *z*) are depicted in grey dotted lines. Only H atoms involved in the C—H⋯O inter­actions and only one component of the disordered perchlorate anion and water mol­ecule are shown.

The non-coordinating perchlorate anion connects, *via* a net of weak hydrogen bonds, three {Ba[Co(H_-2_ **L1**)](ClO_4_)(H_2_O)}^+^ cationic fragments belonging to two different 1D-CPs wave-like disposed along the *b* axis (Fig. 6 ▸).

## Database survey   

Five structures containing **L1** were found in a search of the CSD (Version 5.38, May 2017; Groom *et al.*, 2016 ▸), three of them containing Co^II^: a hetero-trinuclear Gd^III^–Co^II^–Gd^III^ dimer, a hetero-dinuclear Na^I^–Co^II^ complex and a Co^II^ complex (Benelli *et al.*, 2013 ▸; Amatori *et al.*, 2012 ▸; Borgogelli *et al.*, 2013 ▸). In addition, our group recently published the corresponding hetero-trinuclear Eu–Co–Eu dimer (Rossi *et al.*, 2017 ▸).

A general search for structures containing both Co^II^ and Ba^II^ ions revealed 61 hits, 20 of which are polymeric structures formed by organic ligands containing both oxygen and nitro­gen donor atoms and only two being 1D-CPs. It is noteworthy that none of the 20 structures contains either macrocyclic ligands or an N_4_O_2_ donor set around the Co^II^ ion. In eight out of those 20 polymeric structures, the Ba^II^ and Co^II^ ions are bridged by oxygen atoms and ten out of 20 show oxygen-bridged Ba^II^ ions (only eight forming an infinite chain). Finally, only six out of the 20 polymeric structures contain both oxygen-bridged Ba^II^ ions and oxygen-bridged Ba^II^ and Co^II^ ions.

All these data suggest that structures containing both oxygen-bridged Ba^II^ ions and oxygen-bridged Ba^II^ and Co^II^ ions are not common and that no Ba^II^-based 1D-CPs formed by macrocyclic ligands and containing Co^II^ ions surrounded by an N_4_O_2_ donor set are present in the CSD.

## Synthesis and crystallization   

Compound **L1** was obtained following the synthetic procedure previously reported (Amatori *et al.*, 2012 ▸).

To obtain the Ba^II^-based Co^II^-containing 1D-CP of **L1**, {{Ba[Co(H_–2_ **L1**)](ClO_4_)(H_2_O)}·ClO_4_}_*n*_, 0.1 mmol of CoCl_2_· 6H_2_O in water (10 mL) were added to an aqueous solution (20 mL) containing 0.1 mmol of **L1**·3HClO_4_·H_2_O. The solution was adjusted to pH 7 with 0.1 *M* N(CH_3_)_4_OH and then 0.05 mmol of BaCl_2_· 2H_2_O were added. The solution was saturated with NaClO_4_. The Ba^II^–Co^II^ 1D-CP of **L1** quickly precipitated as a microcrystalline pink solid. Crystals suitable for X-ray analysis were instead obtained by slow evaporation of a more diluted aqueous solution.

## Refinement   

Crystal data, data collection and structure refinement details are summarized in Table 3 ▸. All hydrogen atoms of the macrocycle were positioned geometrically and refined as riding with C—H = 0.95–0.99 Å with *U*_iso_(H) = 1.5*U*_eq_(C-meth­yl) and = 1.2*U*_eq_(C) for other H atoms. Both perchlorate anions are disordered, all oxygen and chlorine atoms were set in double positions [anion 1: Cl1*A*/*B*, O11*A*/*B*, O12*A*/*B*, O13*A*/*B*, O14*A*/*B*, occupancy factor: 0.40 (3) and 0.60 (3); anion 2: Cl2*A*/*B*, O21*A*/*B*, O22*A*/*B*, O23*A*/*B*, O24*A*/*B*, occupancy factor: 0.78 (3) and 0.22 (3)]. The water mol­ecule is disordered over three positions [SUMP command was used, occupancies 0.49 (3), 0.27 (3) and 0.24 (3)], the hydrogen atoms were not found in the Fourier-difference map and they were not introduced in the refinement. All non-hydrogen atoms were anisotropically refined: as for the disordered perchlorate anions, the SIMU instruction was used to restrain the anisotropic displacement parameters of the disordered atoms, while the ISOR instruction was used to model the disordered water oxygen atoms.

## Supplementary Material

Crystal structure: contains datablock(s) global, I. DOI: 10.1107/S2056989017015638/bq2404sup1.cif

Structure factors: contains datablock(s) I. DOI: 10.1107/S2056989017015638/bq2404Isup2.hkl

Supporting information file. DOI: 10.1107/S2056989017015638/bq2404Isup3.mol

CCDC reference: 1582341

Additional supporting information:  crystallographic information; 3D view; checkCIF report

## Figures and Tables

**Table 1:** Selected bond lengths and angles (Å, °)

Co1—N1	2.199 (3)
Co1—N2	2.414 (3)
Co1—N3	2.220 (4)
Co1—N4	2.344 (3)
Co1—O1	2.044 (3)
Co1—O4	2.075 (3)
Ba1—O1	2.688 (3)
Ba1—O2	2.861 (3)
Ba1—O4	2.690 (3)
Ba1—O5	2.814 (3)
Ba1—O1*W*	2.774 (14)/2.75 (2)/2.972 (15)^*a*^
Ba1—O11	2.853 (19)/3.154 (13)^*b*^
Ba1—O12	2.955 (18)/2.863 (12)^*b*^
N1⋯N3	3.903 (5)
N2⋯N4	4.164 (5)
Ba1⋯Ba1^i^	4.9123 (4)
Ba1⋯O2^i^	2.860 (3)
O2⋯O2^i^	2.932 (4)
Ba1⋯Ba1^ii^	4.8443 (4)
Ba1⋯O5^ii^	2.900 (3)
O5⋯O5^ii^	3.033 (4)
Ba1^i^⋯Ba1^ii^	8.8965 (4)
	
Ba1—O1—Co1	113.82 (12)
Ba1—O4—Co1	112.64 (11)
Ba1—O2—Ba1^i^	118.34 (10)
Ba1—O5—Ba1^ii^	115.92 (10)

Symmetry codes: (i) = −*x* + 1, −*y*, −*z*; (ii) = −*x* + 2, −*y*, −*z*. Notes: (*a*) the values refer to O1*WA*/*B*/*C* atoms, respectively; (*b*) the values refer to the *A*/*B* oxygen atoms, respectively, of the disordered perchlorate anion (see *Refinement* section).

**Table 2:** Hydrogen-bond geometry (Å, °)

*D*—H⋯	*D*—H	H⋯*A*	*D*⋯*A*	*D*—H⋯*A*
C8—H8*A*⋯O21	0.99	2.91/2.62	3.877 (12)/3.60 (3)	165.3/172.3
C3—H3*A*⋯O22^iii^	0.99	2.65/2.48	3.577 (11)/3.46 (3)	155.7/166.7
C8—H8*B*⋯O13^iv^	0.99	2.49/2.59	3.46 (2)/3.524 (15)	168.5/156.6
C22—H22⋯O21^v^	0.95	2.62/2.66	3.482 (11)/3.51 (3)	151.4/149.2
C22—H22⋯O23^v^	0.95	2.57/2.54	3.455 (11)/3.39 (4)	155.1/149.9

Symmetry codes: (iii) −*x* + 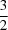, *y* − 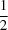, −*z* + 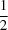; (iv) *x* + 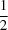, −*y* + 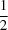, *z* − 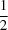; (v) *x* − 1, *y*, *z*. Note: the first and second values for each entry refer to the *A* and *B* oxygen atoms, respectively, of the disordered perchlorate anion (see *Refinement* section).

**Table 3:** Experimental details

Crystal data	
Chemical formula	[BaCo(C_22_H_28_N_4_O_6_)(ClO_4_)(H_2_O)]·ClO_4_
*M*_r_	857.67
Crystal system, space group	Monoclinic, *P*2_1_/*n*
Temperature (K)	120
*a*, *b*, *c* (Å)	8.8965 (2), 18.0995 (4), 19.0103 (6)
β (°)	94.572 (2)
*V* (Å^3^)	3051.34 (14)
*Z*	4
Radiation type	Mo *K*α
μ (mm^−1^)	2.08
Crystal size (mm)	0.45 × 0.38 × 0.27

Data collection	
Diffractometer	Rigaku OD Xcalibur, Sapphire3
Absorption correction	Multi-scan (*CrysAlis PRO*; Rigaku OD, 2015 ▸). Empirical absorption correction using spherical harmonics, implemented in SCALE3 ABSPACK scaling algorithm.
*T*_min_, *T*_max_	0.884, 1.000
No. of measured, independent and observed [*I* > 2σ(*I*)] reflections	15496, 6966, 5315
*R*_int_	0.037
(sin θ/λ)_max_ (Å^−1^)	0.682

Refinement	
*R*[*F* ^2^ > 2σ(*F* ^2^)], *wR*(*F* ^2^), *S*	0.041, 0.103, 1.06
No. of reflections	6966
No. of parameters	519
No. of restraints	133
H-atom treatment	H-atom parameters constrained
Δρ_max_, Δρ_min_ (e Å^−3^)	1.00, −0.72

Computer programs: *CrysAlis PRO* (Rigaku OD, 2015 ▸), *SIR2014* (Burla, 2015 ▸), *SHELXL2014*/7 (Sheldrick, 2015 ▸).

## supplementary crystallographic information

### Crystal data

**Table:**

[BaCo(C_22_H_28_N_4_O_6_)(ClO_4_)(H_2_O)]ClO_4_	*F*(000) = 1708
*M**_r_* = 857.67	*D*_x_ = 1.867 Mg m^−^^3^
Monoclinic, *P*2_1_/*n*	Mo *K*α radiation, λ = 0.71073 Å
*a* = 8.8965 (2) Å	Cell parameters from 4989 reflections
*b* = 18.0995 (4) Å	θ = 2.2–27.7°
*c* = 19.0103 (6) Å	µ = 2.08 mm^−^^1^
β = 94.572 (2)°	*T* = 120 K
*V* = 3051.34 (14) Å^3^	Prism, pink
*Z* = 4	0.45 × 0.38 × 0.27 mm

### Data collection

**Table:**

Rigaku OD Xcalibur, Sapphire3 diffractometer	6966 independent reflections
Radiation source: fine-focus sealed X-ray tube, Enhance (Mo) X-ray Source	5315 reflections with *I* > 2σ(*I*)
Graphite monochromator	*R*_int_ = 0.037
Detector resolution: 16.4547 pixels mm^-1^	θ_max_ = 29.0°, θ_min_ = 2.2°
ω scans	*h* = −12→11
Absorption correction: multi-scan (CrysAlisPro; Rigaku OD, 2015) Empirical absorption correction using spherical harmonics, implemented in SCALE3 ABSPACK scaling algorithm.	*k* = −22→22
*T*_min_ = 0.884, *T*_max_ = 1.000	*l* = −22→24
15496 measured reflections	

### Refinement

**Table:**

Refinement on *F*^2^	133 restraints
Least-squares matrix: full	Hydrogen site location: inferred from neighbouring sites
*R*[*F*^2^ > 2σ(*F*^2^)] = 0.041	H-atom parameters constrained
*wR*(*F*^2^) = 0.103	*w* = 1/[σ^2^(*F*_o_^2^) + (0.037*P*)^2^ + 2.9358*P*] where *P* = (*F*_o_^2^ + 2*F*_c_^2^)/3
*S* = 1.06	(Δ/σ)_max_ = 0.001
6966 reflections	Δρ_max_ = 1.00 e Å^−^^3^
519 parameters	Δρ_min_ = −0.72 e Å^−^^3^

### Special details

**Table:**

Geometry. All esds (except the esd in the dihedral angle between two l.s. planes) are estimated using the full covariance matrix. The cell esds are taken into account individually in the estimation of esds in distances, angles and torsion angles; correlations between esds in cell parameters are only used when they are defined by crystal symmetry. An approximate (isotropic) treatment of cell esds is used for estimating esds involving l.s. planes.

### Fractional atomic coordinates and isotropic or equivalent isotropic displacement parameters (Å^2^)

**Table:**

	*x*	*y*	*z*	*U*_iso_*/*U*_eq_	Occ. (<1)
Ba1	0.75388 (3)	0.05421 (2)	0.01055 (2)	0.03237 (9)	
Co1	0.75301 (6)	0.26481 (3)	0.07072 (3)	0.02836 (14)	
O1	0.6344 (3)	0.19069 (15)	0.00703 (16)	0.0351 (7)	
N1	0.9308 (4)	0.33476 (19)	0.03199 (19)	0.0336 (8)	
C1	0.5130 (6)	0.3782 (3)	0.1078 (3)	0.0545 (14)	
H1A	0.4189	0.3897	0.1300	0.065*	
H1B	0.5870	0.4177	0.1210	0.065*	
O2	0.4340 (3)	0.07423 (16)	−0.00434 (18)	0.0400 (8)	
N2	0.6147 (4)	0.35215 (19)	−0.00548 (19)	0.0350 (8)	
C2	0.4817 (5)	0.3772 (3)	0.0300 (3)	0.0461 (12)	
H2A	0.3957	0.3438	0.0172	0.055*	
H2B	0.4530	0.4275	0.0133	0.055*	
O3	0.3318 (3)	0.26420 (17)	−0.11008 (16)	0.0374 (7)	
N3	0.5736 (4)	0.3060 (2)	0.1354 (2)	0.0389 (9)	
C3	0.7199 (5)	0.4133 (3)	−0.0140 (3)	0.0445 (11)	
H3A	0.7282	0.4440	0.0292	0.053*	
H3B	0.6818	0.4449	−0.0541	0.053*	
O4	0.8695 (3)	0.16663 (15)	0.09010 (15)	0.0340 (7)	
N4	0.8905 (4)	0.30110 (18)	0.17530 (19)	0.0324 (8)	
C4	0.8729 (5)	0.3827 (3)	−0.0275 (3)	0.0434 (11)	
H4A	0.8650	0.3537	−0.0718	0.052*	
H4B	0.9442	0.4239	−0.0330	0.052*	
O5	1.0650 (3)	0.05816 (16)	0.05118 (17)	0.0406 (8)	
C5	0.9983 (5)	0.3822 (2)	0.0890 (3)	0.0422 (11)	
H5A	0.9293	0.4240	0.0962	0.051*	
H5B	1.0941	0.4029	0.0746	0.051*	
O6	1.1536 (4)	0.16710 (18)	0.23669 (17)	0.0462 (8)	
C6	1.0284 (5)	0.3412 (3)	0.1570 (3)	0.0414 (11)	
H6A	1.1114	0.3055	0.1527	0.050*	
H6B	1.0601	0.3764	0.1952	0.050*	
C7	0.7907 (5)	0.3517 (3)	0.2110 (3)	0.0426 (11)	
H7A	0.7868	0.4003	0.1870	0.051*	
H7B	0.8308	0.3593	0.2606	0.051*	
C8	0.6351 (5)	0.3191 (3)	0.2090 (3)	0.0442 (12)	
H8A	0.6387	0.2719	0.2353	0.053*	
H8B	0.5678	0.3533	0.2324	0.053*	
C9	0.4532 (6)	0.2506 (3)	0.1367 (3)	0.0589 (15)	
H9A	0.4106	0.2409	0.0885	0.088*	
H9B	0.4947	0.2047	0.1576	0.088*	
H9C	0.3738	0.2691	0.1650	0.088*	
C10	1.0466 (5)	0.2848 (3)	0.0060 (3)	0.0500 (13)	
H10A	1.0003	0.2536	−0.0319	0.075*	
H10B	1.1274	0.3143	−0.0121	0.075*	
H10C	1.0886	0.2536	0.0448	0.075*	
C11	0.5658 (5)	0.3217 (2)	−0.0762 (2)	0.0395 (10)	
H11A	0.5112	0.3604	−0.1049	0.047*	
H11B	0.6556	0.3072	−0.1006	0.047*	
C12	0.4661 (5)	0.2566 (2)	−0.0707 (2)	0.0318 (9)	
C13	0.5033 (4)	0.1960 (2)	−0.0315 (2)	0.0316 (9)	
C14	0.3993 (4)	0.1344 (2)	−0.0339 (2)	0.0325 (9)	
C15	0.2580 (4)	0.1477 (2)	−0.0733 (2)	0.0310 (9)	
H15	0.1815	0.1109	−0.0739	0.037*	
C16	0.2308 (5)	0.2098 (3)	−0.1090 (2)	0.0367 (10)	
H16	0.1358	0.2158	−0.1349	0.044*	
C17	0.9321 (5)	0.2400 (2)	0.2241 (2)	0.0404 (11)	
H17A	0.9863	0.2604	0.2673	0.048*	
H17B	0.8390	0.2160	0.2380	0.048*	
C18	1.0276 (5)	0.1841 (2)	0.1936 (2)	0.0354 (10)	
C19	0.9936 (4)	0.1509 (2)	0.1305 (2)	0.0307 (9)	
C20	1.0956 (5)	0.0936 (2)	0.1072 (2)	0.0364 (10)	
C21	1.2276 (5)	0.0819 (3)	0.1537 (3)	0.0479 (12)	
H21	1.3017	0.0479	0.1404	0.058*	
C22	1.2496 (6)	0.1168 (3)	0.2147 (3)	0.0536 (14)	
H22	1.3377	0.1056	0.2443	0.064*	
Cl1A	0.800 (2)	0.1249 (9)	−0.1620 (10)	0.047 (2)	0.40 (3)
O11A	0.658 (2)	0.0849 (10)	−0.1333 (10)	0.047 (3)	0.40 (3)
O12A	0.9088 (18)	0.1007 (11)	−0.1140 (9)	0.052 (3)	0.40 (3)
O13A	0.860 (3)	0.0808 (10)	−0.2136 (12)	0.063 (4)	0.40 (3)
O14A	0.810 (3)	0.2057 (12)	−0.1686 (12)	0.074 (4)	0.40 (3)
Cl1B	0.7844 (14)	0.1280 (7)	−0.1622 (6)	0.0479 (16)	0.60 (3)
O11B	0.6498 (15)	0.1036 (8)	−0.1448 (7)	0.051 (2)	0.60 (3)
O12B	0.8977 (12)	0.1255 (8)	−0.1006 (6)	0.056 (2)	0.60 (3)
O13B	0.8144 (15)	0.0963 (8)	−0.2287 (6)	0.064 (3)	0.60 (3)
O14B	0.750 (2)	0.2000 (8)	−0.1816 (7)	0.076 (3)	0.60 (3)
Cl2A	0.6555 (9)	0.0780 (5)	0.3226 (4)	0.0424 (12)	0.78 (3)
O21A	0.5765 (11)	0.1464 (5)	0.3243 (6)	0.066 (2)	0.78 (3)
O22A	0.7030 (10)	0.0638 (6)	0.3946 (4)	0.062 (2)	0.78 (3)
O23A	0.5501 (11)	0.0220 (4)	0.2981 (6)	0.065 (2)	0.78 (3)
O24A	0.7821 (9)	0.0858 (7)	0.2801 (4)	0.081 (3)	0.78 (3)
Cl2B	0.661 (4)	0.073 (2)	0.317 (2)	0.060 (5)	0.22 (3)
O21B	0.610 (4)	0.1450 (16)	0.2989 (19)	0.053 (6)	0.22 (3)
O22B	0.733 (4)	0.0437 (17)	0.381 (2)	0.065 (5)	0.22 (3)
O23B	0.571 (4)	0.027 (2)	0.272 (2)	0.066 (5)	0.22 (3)
O24B	0.783 (3)	0.049 (2)	0.2882 (15)	0.071 (5)	0.22 (3)
O1WA	0.6542 (14)	0.0192 (9)	0.1411 (7)	0.079 (4)	0.493 (3)
O1WB	0.721 (2)	0.0144 (13)	0.1481 (12)	0.054 (5)	0.268 (3)
O1WC	0.842 (2)	−0.0011 (8)	0.1552 (8)	0.058 (4)	0.239 (3)

### Atomic displacement parameters (Å^2^)

**Table:**

	*U*^11^	*U*^22^	*U*^33^	*U*^12^	*U*^13^	*U*^23^
Ba1	0.02904 (15)	0.02521 (14)	0.04255 (17)	0.00132 (10)	0.00089 (10)	−0.00540 (11)
Co1	0.0275 (3)	0.0241 (3)	0.0336 (3)	0.0011 (2)	0.0035 (2)	−0.0029 (2)
O1	0.0309 (15)	0.0251 (15)	0.0476 (19)	0.0022 (12)	−0.0083 (13)	−0.0040 (13)
N1	0.0362 (19)	0.0289 (19)	0.037 (2)	−0.0018 (15)	0.0072 (15)	−0.0018 (16)
C1	0.052 (3)	0.049 (3)	0.065 (4)	0.019 (2)	0.015 (3)	−0.007 (3)
O2	0.0332 (16)	0.0273 (16)	0.058 (2)	−0.0005 (12)	−0.0026 (14)	0.0005 (15)
N2	0.0336 (19)	0.031 (2)	0.040 (2)	0.0002 (15)	0.0013 (15)	−0.0011 (16)
C2	0.041 (3)	0.041 (3)	0.056 (3)	0.013 (2)	0.005 (2)	−0.007 (2)
O3	0.0348 (16)	0.0409 (18)	0.0354 (18)	0.0054 (13)	−0.0043 (13)	0.0019 (14)
N3	0.0327 (19)	0.045 (2)	0.040 (2)	0.0041 (17)	0.0069 (16)	−0.0030 (18)
C3	0.057 (3)	0.029 (2)	0.048 (3)	−0.001 (2)	0.000 (2)	0.008 (2)
O4	0.0332 (16)	0.0265 (15)	0.0403 (18)	0.0056 (12)	−0.0102 (12)	−0.0067 (13)
N4	0.0350 (19)	0.0272 (18)	0.035 (2)	0.0008 (15)	0.0057 (15)	−0.0068 (15)
C4	0.047 (3)	0.041 (3)	0.043 (3)	−0.011 (2)	0.008 (2)	0.007 (2)
O5	0.0372 (17)	0.0359 (18)	0.047 (2)	0.0083 (13)	−0.0064 (14)	−0.0124 (15)
C5	0.039 (3)	0.033 (2)	0.054 (3)	−0.008 (2)	0.002 (2)	−0.003 (2)
O6	0.051 (2)	0.0442 (19)	0.040 (2)	0.0051 (15)	−0.0151 (15)	−0.0055 (16)
C6	0.037 (2)	0.036 (2)	0.050 (3)	−0.005 (2)	−0.001 (2)	−0.005 (2)
C7	0.045 (3)	0.041 (3)	0.043 (3)	0.003 (2)	0.007 (2)	−0.015 (2)
C8	0.048 (3)	0.041 (3)	0.046 (3)	0.006 (2)	0.016 (2)	−0.008 (2)
C9	0.047 (3)	0.072 (4)	0.059 (4)	−0.016 (3)	0.016 (3)	−0.007 (3)
C10	0.043 (3)	0.051 (3)	0.059 (3)	0.002 (2)	0.020 (2)	−0.008 (3)
C11	0.045 (3)	0.034 (2)	0.039 (3)	0.000 (2)	0.001 (2)	0.002 (2)
C12	0.034 (2)	0.033 (2)	0.028 (2)	−0.0013 (18)	−0.0015 (17)	0.0001 (18)
C13	0.027 (2)	0.030 (2)	0.038 (2)	0.0033 (17)	−0.0002 (17)	−0.0061 (18)
C14	0.030 (2)	0.033 (2)	0.035 (2)	0.0061 (17)	0.0020 (17)	−0.0075 (19)
C15	0.025 (2)	0.037 (2)	0.029 (2)	−0.0004 (17)	−0.0038 (16)	−0.0079 (18)
C16	0.032 (2)	0.047 (3)	0.031 (2)	0.003 (2)	−0.0022 (17)	−0.003 (2)
C17	0.048 (3)	0.038 (3)	0.035 (3)	0.001 (2)	0.001 (2)	−0.003 (2)
C18	0.034 (2)	0.032 (2)	0.039 (3)	0.0022 (18)	−0.0061 (18)	0.0016 (19)
C19	0.031 (2)	0.026 (2)	0.034 (2)	−0.0013 (16)	−0.0025 (17)	−0.0028 (18)
C20	0.035 (2)	0.029 (2)	0.043 (3)	0.0009 (18)	−0.0058 (19)	−0.002 (2)
C21	0.040 (3)	0.037 (3)	0.064 (4)	0.010 (2)	−0.013 (2)	−0.011 (2)
C22	0.044 (3)	0.049 (3)	0.063 (4)	0.012 (2)	−0.024 (2)	−0.007 (3)
Cl1A	0.049 (4)	0.040 (4)	0.054 (4)	−0.001 (3)	0.010 (3)	0.013 (3)
O11A	0.047 (5)	0.039 (5)	0.055 (5)	−0.001 (4)	0.003 (4)	0.015 (4)
O12A	0.050 (5)	0.049 (5)	0.058 (5)	0.007 (4)	0.003 (4)	0.005 (4)
O13A	0.065 (7)	0.063 (6)	0.062 (7)	0.013 (5)	0.022 (5)	0.006 (5)
O14A	0.073 (8)	0.053 (7)	0.093 (8)	0.006 (7)	−0.019 (7)	0.024 (6)
Cl1B	0.049 (3)	0.057 (3)	0.037 (2)	0.0143 (18)	0.0014 (18)	0.0026 (18)
O11B	0.042 (3)	0.064 (5)	0.049 (4)	0.011 (4)	0.008 (3)	0.006 (4)
O12B	0.049 (3)	0.066 (4)	0.051 (4)	0.009 (4)	−0.006 (3)	0.001 (4)
O13B	0.058 (5)	0.100 (6)	0.035 (4)	0.024 (5)	0.006 (4)	−0.002 (4)
O14B	0.081 (7)	0.063 (5)	0.080 (6)	0.004 (6)	−0.018 (5)	0.025 (4)
Cl2A	0.0529 (19)	0.035 (2)	0.0386 (17)	−0.0050 (18)	−0.0023 (13)	0.0003 (14)
O21A	0.076 (5)	0.045 (3)	0.074 (5)	0.008 (3)	−0.011 (4)	−0.010 (4)
O22A	0.074 (4)	0.065 (5)	0.046 (4)	0.015 (3)	−0.001 (3)	0.007 (3)
O23A	0.082 (4)	0.034 (3)	0.074 (5)	−0.020 (3)	−0.022 (4)	0.006 (4)
O24A	0.097 (4)	0.070 (6)	0.083 (4)	−0.021 (4)	0.046 (3)	−0.011 (4)
Cl2B	0.075 (8)	0.034 (6)	0.069 (9)	0.014 (5)	0.002 (6)	−0.003 (5)
O21B	0.071 (10)	0.020 (8)	0.066 (12)	−0.001 (7)	−0.002 (9)	0.004 (8)
O22B	0.086 (10)	0.039 (9)	0.068 (10)	0.012 (7)	−0.003 (8)	−0.002 (7)
O23B	0.086 (9)	0.043 (9)	0.069 (11)	0.000 (7)	0.008 (8)	−0.007 (8)
O24B	0.091 (9)	0.046 (8)	0.076 (10)	0.009 (7)	0.012 (7)	−0.006 (7)
O1WA	0.100 (8)	0.095 (7)	0.042 (5)	0.000 (7)	0.005 (6)	−0.007 (5)
O1WB	0.067 (9)	0.056 (8)	0.038 (8)	−0.005 (8)	0.004 (8)	0.001 (6)
O1WC	0.077 (8)	0.045 (7)	0.052 (8)	−0.012 (6)	0.010 (6)	−0.012 (6)

### Geometric parameters (Å, º)

**Table:**

Ba1—O1	2.688 (3)	C5—H5B	0.9900
Ba1—O4	2.690 (3)	O6—C22	1.339 (6)
Ba1—O1WB	2.75 (2)	O6—C18	1.370 (5)
Ba1—O1WA	2.774 (14)	C6—H6A	0.9900
Ba1—O5	2.814 (3)	C6—H6B	0.9900
Ba1—O11A	2.853 (19)	C7—C8	1.503 (6)
Ba1—O2^i^	2.860 (3)	C7—H7A	0.9900
Ba1—O2	2.861 (3)	C7—H7B	0.9900
Ba1—O12B	2.863 (10)	C8—H8A	0.9900
Ba1—O5^ii^	2.901 (3)	C8—H8B	0.9900
Ba1—O12A	2.955 (15)	C9—H9A	0.9800
Ba1—O1WC	2.972 (17)	C9—H9B	0.9800
Co1—O1	2.044 (3)	C9—H9C	0.9800
Co1—O4	2.075 (3)	C10—H10A	0.9800
Co1—N1	2.199 (3)	C10—H10B	0.9800
Co1—N3	2.220 (3)	C10—H10C	0.9800
Co1—N4	2.344 (4)	C11—C12	1.484 (6)
Co1—N2	2.414 (4)	C11—H11A	0.9900
O1—C13	1.330 (5)	C11—H11B	0.9900
N1—C5	1.472 (6)	C12—C13	1.351 (6)
N1—C10	1.484 (5)	C13—C14	1.448 (6)
N1—C4	1.485 (6)	C14—C15	1.432 (5)
C1—C2	1.484 (7)	C15—C16	1.326 (6)
C1—N3	1.492 (6)	C15—H15	0.9500
C1—H1A	0.9900	C16—H16	0.9500
C1—H1B	0.9900	C17—C18	1.471 (6)
O2—C14	1.252 (5)	C17—H17A	0.9900
O2—Ba1^i^	2.860 (3)	C17—H17B	0.9900
N2—C3	1.466 (6)	C18—C19	1.353 (6)
N2—C2	1.479 (5)	C19—C20	1.469 (6)
N2—C11	1.486 (6)	C20—C21	1.429 (6)
C2—H2A	0.9900	C21—C22	1.320 (7)
C2—H2B	0.9900	C21—H21	0.9500
O3—C16	1.334 (5)	C22—H22	0.9500
O3—C12	1.366 (5)	Cl1A—O12A	1.35 (2)
N3—C9	1.470 (6)	Cl1A—O13A	1.40 (2)
N3—C8	1.481 (6)	Cl1A—O14A	1.47 (3)
C3—C4	1.511 (6)	Cl1A—O11A	1.59 (2)
C3—H3A	0.9900	Cl1B—O11B	1.342 (18)
C3—H3B	0.9900	Cl1B—O14B	1.383 (17)
O4—C19	1.325 (5)	Cl1B—O13B	1.433 (15)
N4—C17	1.471 (5)	Cl1B—O12B	1.484 (15)
N4—C7	1.477 (5)	Cl2A—O22A	1.423 (11)
N4—C6	1.490 (5)	Cl2A—O21A	1.425 (12)
C4—H4A	0.9900	Cl2A—O23A	1.434 (10)
C4—H4B	0.9900	Cl2A—O24A	1.444 (11)
O5—C20	1.254 (5)	Cl2B—O24B	1.33 (5)
O5—Ba1^ii^	2.901 (3)	Cl2B—O23B	1.40 (4)
C5—C6	1.497 (6)	Cl2B—O21B	1.42 (4)
C5—H5A	0.9900	Cl2B—O22B	1.43 (5)
			
O1—Ba1—O4	56.75 (8)	N1—C4—H4A	109.6
O1—Ba1—O1WB	101.1 (5)	C3—C4—H4A	109.6
O4—Ba1—O1WB	74.2 (5)	N1—C4—H4B	109.6
O1—Ba1—O1WA	94.5 (3)	C3—C4—H4B	109.6
O4—Ba1—O1WA	78.8 (3)	H4A—C4—H4B	108.1
O1—Ba1—O5	111.22 (8)	C20—O5—Ba1	112.8 (3)
O4—Ba1—O5	60.20 (8)	C20—O5—Ba1^ii^	127.8 (3)
O1WB—Ba1—O5	85.5 (4)	Ba1—O5—Ba1^ii^	115.92 (10)
O1WA—Ba1—O5	97.9 (3)	N1—C5—C6	112.4 (4)
O1—Ba1—O11A	73.1 (4)	N1—C5—H5A	109.1
O4—Ba1—O11A	117.6 (4)	C6—C5—H5A	109.1
O1WA—Ba1—O11A	144.0 (5)	N1—C5—H5B	109.1
O5—Ba1—O11A	118.1 (4)	C6—C5—H5B	109.1
O1—Ba1—O2^i^	121.15 (8)	H5A—C5—H5B	107.9
O4—Ba1—O2^i^	146.50 (9)	C22—O6—C18	118.5 (4)
O1WB—Ba1—O2^i^	73.8 (5)	N4—C6—C5	110.4 (4)
O1WA—Ba1—O2^i^	67.9 (3)	N4—C6—H6A	109.6
O5—Ba1—O2^i^	126.20 (8)	C5—C6—H6A	109.6
O11A—Ba1—O2^i^	89.4 (4)	N4—C6—H6B	109.6
O1—Ba1—O2	59.51 (8)	C5—C6—H6B	109.6
O4—Ba1—O2	107.01 (8)	H6A—C6—H6B	108.1
O1WB—Ba1—O2	87.0 (5)	N4—C7—C8	109.4 (4)
O1WA—Ba1—O2	74.4 (3)	N4—C7—H7A	109.8
O5—Ba1—O2	166.61 (9)	C8—C7—H7A	109.8
O11A—Ba1—O2	70.2 (4)	N4—C7—H7B	109.8
O2^i^—Ba1—O2	61.66 (10)	C8—C7—H7B	109.8
O1—Ba1—O12B	76.5 (3)	H7A—C7—H7B	108.2
O4—Ba1—O12B	84.3 (3)	N3—C8—C7	110.9 (4)
O1WB—Ba1—O12B	155.1 (5)	N3—C8—H8A	109.5
O5—Ba1—O12B	72.8 (2)	C7—C8—H8A	109.5
O2^i^—Ba1—O12B	129.0 (3)	N3—C8—H8B	109.5
O2—Ba1—O12B	111.7 (2)	C7—C8—H8B	109.5
O1—Ba1—O5^ii^	149.97 (9)	H8A—C8—H8B	108.1
O4—Ba1—O5^ii^	123.92 (8)	N3—C9—H9A	109.5
O1WB—Ba1—O5^ii^	107.8 (5)	N3—C9—H9B	109.5
O1WA—Ba1—O5^ii^	115.4 (3)	H9A—C9—H9B	109.5
O5—Ba1—O5^ii^	64.08 (10)	N3—C9—H9C	109.5
O11A—Ba1—O5^ii^	83.3 (4)	H9A—C9—H9C	109.5
O2^i^—Ba1—O5^ii^	75.82 (9)	H9B—C9—H9C	109.5
O2—Ba1—O5^ii^	128.99 (9)	N1—C10—H10A	109.5
O12B—Ba1—O5^ii^	73.8 (3)	N1—C10—H10B	109.5
O1—Ba1—O12A	85.8 (4)	H10A—C10—H10B	109.5
O4—Ba1—O12A	93.0 (4)	N1—C10—H10C	109.5
O1WA—Ba1—O12A	169.9 (4)	H10A—C10—H10C	109.5
O5—Ba1—O12A	72.6 (3)	H10B—C10—H10C	109.5
O11A—Ba1—O12A	45.6 (5)	C12—C11—N2	111.3 (4)
O2^i^—Ba1—O12A	120.5 (4)	C12—C11—H11A	109.4
O2—Ba1—O12A	114.1 (3)	N2—C11—H11A	109.4
O5^ii^—Ba1—O12A	64.3 (4)	C12—C11—H11B	109.4
O1—Ba1—O1WC	114.0 (3)	N2—C11—H11B	109.4
O4—Ba1—O1WC	71.0 (3)	H11A—C11—H11B	108.0
O5—Ba1—O1WC	64.8 (3)	C13—C12—O3	123.3 (4)
O2^i^—Ba1—O1WC	82.8 (3)	C13—C12—C11	124.2 (4)
O2—Ba1—O1WC	108.7 (3)	O3—C12—C11	112.5 (4)
O5^ii^—Ba1—O1WC	91.4 (3)	O1—C13—C12	121.8 (4)
O1—Co1—O4	76.70 (11)	O1—C13—C14	119.4 (4)
O1—Co1—N1	122.08 (13)	C12—C13—C14	118.7 (4)
O4—Co1—N1	100.96 (12)	O2—C14—C15	123.7 (4)
O1—Co1—N3	100.86 (13)	O2—C14—C13	121.5 (4)
O4—Co1—N3	124.07 (13)	C15—C14—C13	114.8 (4)
N1—Co1—N3	124.08 (13)	C16—C15—C14	121.8 (4)
O1—Co1—N4	153.71 (12)	C16—C15—H15	119.1
O4—Co1—N4	82.53 (11)	C14—C15—H15	119.1
N1—Co1—N4	77.40 (13)	C15—C16—O3	122.6 (4)
N3—Co1—N4	77.62 (12)	C15—C16—H16	118.7
O1—Co1—N2	81.95 (12)	O3—C16—H16	118.7
O4—Co1—N2	152.96 (12)	C18—C17—N4	113.1 (4)
N1—Co1—N2	76.58 (12)	C18—C17—H17A	109.0
N3—Co1—N2	75.99 (13)	N4—C17—H17A	109.0
N4—Co1—N2	122.15 (12)	C18—C17—H17B	109.0
C13—O1—Co1	131.9 (2)	N4—C17—H17B	109.0
C13—O1—Ba1	114.1 (2)	H17A—C17—H17B	107.8
Co1—O1—Ba1	113.82 (11)	C19—C18—O6	123.0 (4)
C5—N1—C10	110.3 (4)	C19—C18—C17	124.1 (4)
C5—N1—C4	108.4 (3)	O6—C18—C17	112.9 (4)
C10—N1—C4	108.1 (4)	O4—C19—C18	122.3 (4)
C5—N1—Co1	110.6 (3)	O4—C19—C20	118.9 (4)
C10—N1—Co1	107.3 (3)	C18—C19—C20	118.7 (4)
C4—N1—Co1	112.1 (3)	O5—C20—C21	124.2 (4)
C2—C1—N3	112.0 (4)	O5—C20—C19	121.3 (4)
C2—C1—H1A	109.2	C21—C20—C19	114.5 (4)
N3—C1—H1A	109.2	C22—C21—C20	122.0 (4)
C2—C1—H1B	109.2	C22—C21—H21	119.0
N3—C1—H1B	109.2	C20—C21—H21	119.0
H1A—C1—H1B	107.9	C21—C22—O6	123.1 (4)
C14—O2—Ba1^i^	124.5 (3)	C21—C22—H22	118.4
C14—O2—Ba1	111.2 (2)	O6—C22—H22	118.4
Ba1^i^—O2—Ba1	118.33 (10)	O12A—Cl1A—O13A	89.8 (18)
C3—N2—C2	111.1 (4)	O12A—Cl1A—O14A	109.8 (13)
C3—N2—C11	108.9 (4)	O13A—Cl1A—O14A	118.6 (14)
C2—N2—C11	109.8 (3)	O12A—Cl1A—O11A	99.6 (12)
C3—N2—Co1	105.2 (3)	O13A—Cl1A—O11A	109.7 (13)
C2—N2—Co1	108.4 (3)	O14A—Cl1A—O11A	122.2 (15)
C11—N2—Co1	113.5 (3)	O12A—Cl1A—Ba1	52.5 (8)
N2—C2—C1	111.4 (4)	O13A—Cl1A—Ba1	121.1 (11)
N2—C2—H2A	109.3	O14A—Cl1A—Ba1	116.5 (12)
C1—C2—H2A	109.3	O11A—Cl1A—Ba1	51.0 (8)
N2—C2—H2B	109.3	Cl1A—O11A—Ba1	103.3 (10)
C1—C2—H2B	109.3	Cl1A—O12A—Ba1	106.3 (10)
H2A—C2—H2B	108.0	O11B—Cl1B—O14B	101.1 (12)
C16—O3—C12	118.6 (3)	O11B—Cl1B—O13B	108.6 (9)
C9—N3—C8	108.0 (4)	O14B—Cl1B—O13B	101.2 (12)
C9—N3—C1	111.1 (4)	O11B—Cl1B—O12B	111.0 (10)
C8—N3—C1	106.7 (4)	O14B—Cl1B—O12B	111.2 (9)
C9—N3—Co1	109.7 (3)	O13B—Cl1B—O12B	121.5 (12)
C8—N3—Co1	110.5 (2)	O11B—Cl1B—Ba1	61.1 (7)
C1—N3—Co1	110.8 (3)	O14B—Cl1B—Ba1	124.5 (8)
N2—C3—C4	109.5 (4)	O13B—Cl1B—Ba1	134.0 (9)
N2—C3—H3A	109.8	O12B—Cl1B—Ba1	50.2 (5)
C4—C3—H3A	109.8	Cl1B—O11B—Ba1	97.0 (7)
N2—C3—H3B	109.8	Cl1B—O12B—Ba1	106.3 (6)
C4—C3—H3B	109.8	O22A—Cl2A—O21A	104.2 (9)
H3A—C3—H3B	108.2	O22A—Cl2A—O23A	108.7 (6)
C19—O4—Co1	132.0 (2)	O21A—Cl2A—O23A	108.1 (7)
C19—O4—Ba1	115.2 (2)	O22A—Cl2A—O24A	111.8 (8)
Co1—O4—Ba1	112.64 (11)	O21A—Cl2A—O24A	109.6 (6)
C17—N4—C7	107.8 (3)	O23A—Cl2A—O24A	113.9 (9)
C17—N4—C6	110.0 (3)	O24B—Cl2B—O23B	90 (3)
C7—N4—C6	109.9 (3)	O24B—Cl2B—O21B	116 (3)
C17—N4—Co1	114.3 (3)	O23B—Cl2B—O21B	104 (3)
C7—N4—Co1	105.7 (3)	O24B—Cl2B—O22B	85 (3)
C6—N4—Co1	108.8 (3)	O23B—Cl2B—O22B	120 (3)
N1—C4—C3	110.4 (4)	O21B—Cl2B—O22B	131 (3)

Symmetry codes: (i) −*x*+1, −*y*, −*z*; (ii) −*x*+2, −*y*, −*z*.
